# Assessment of Neuromuscular Function in Patients With Prior Cosmetic Procedures: A Case Report

**Published:** 2019-12-03

**Authors:** Nicole K. Le, Daniel Liauw, Sabrina Z. Siddiqui, Kevin M. Donohue

**Affiliations:** ^a^Department of Plastic Surgery, Morsani College of Medicine, University of South Florida, Tampa; ^b^Yale School of Public Health, New Haven, Conn; ^c^University of North Carolina School of Medicine, Chapel Hill; ^d^Texas Tech University Health Sciences Center School of Medicine, Lubbock; ^e^Department of Medicine, Saint Joseph Hospital, Lexington, Ky

**Keywords:** cisatracurium besylate (Nimbex), onabotulinum toxin A (Botox), train-of-four peripheral nerve stimulation, neuromuscular blockade, facial nerve

## Abstract

**Objective:** The use of Botulinum toxin type A (Botox) for cosmetic procedures has become so prevalent that many patients do not always consider it to be a past surgical procedure, and it goes excluded from the medical record. Without the knowledge of prior Botox use, interpretation of facial nerve train-of-four testing may be inaccurate. **Methods:** We describe a 61-year-old woman with a history of multiple cosmetic procedures whose postoperative course was complicated by multiorgan system failure, requiring neuromuscular blockade while on mechanical ventilatory support. **Results:** Gauged by facial nerve stimulation, adequate neuromuscular blockade was assumed. However, patient-ventilator dyssynchrony motivated the decision to move the peripheral nerve stimulator to the ulnar nerve, where muscle twitches were observed. This indicated inadequate paralysis. **Conclusions:** This case report highlights the importance of monitoring neuromuscular function with ulnar nerve testing in patients with a history of cosmetic Botox procedures.

Facial rejuvenation with Botulinum toxin type A (Botox) has increased 797% from 2000 to 2016, with a total of 7,056,255 procedures performed in 2016.^1^ Unfortunately, patients do not always disclose prior Botox procedures during their consultations with their doctors for a number or reasons, particularly as they do not regard it as a relevant medical procedure. This can lead to misinterpretations of train-of-four testing using facial nerves with cleaved synaptosome-associated protein (SNAP-25) caused by a previously unreported use of Botox.^2^ Neuromuscular blocking agents are increasingly used in the treatment of critically ill patients for a variety of reasons. There is increasing evidence that the use of these agents is beneficial as part of a lung-protective strategy in acute respiratory distress syndrome (ARDS).^3^

There have been a few published case reports, dating back to 2006, that describe a finding of incomplete paralysis despite 0 of 4 twitches with facial nerve train-of-four stimulation in patients treated with Botox.^4^^-^7 We present a case where a lack of knowledge of a patient's prior Botox procedure led to suboptimal monitoring of her clinical status.

## METHODS

A 61-year-old, white woman with a history of paroxysmal atrial fibrillation, mitral valve regurgitation, and tricuspid valve regurgitation secondary to atrial septal defect was admitted to the cardiothoracic intensive care unit following mitral valve replacement and atrial septal defect repair. Her postoperative course was complicated by acute encephalopathy with seizure activity, cardiogenic shock, and ARDS. During the course of treatment, she underwent neuromuscular blockade with a cisatracurium besylate (Nimbex) drip as part of a lung-protective strategy often implemented in patients with early ARDS.

## RESULTS

While on ventilatory support, she had several days of documented paralysis, gauged by 0 of 4 twitches on facial nerve stimulation via a train-of-four peripheral nerve stimulator. An observed lack of patient-ventilator synchrony during clinical rounds, despite prior documentation of complete paralysis, prompted the decision to move the peripheral nerve stimulator to the ulnar nerve, where muscle twitches were observed. Cisatracurium was titrated until adequate paralysis was obtained, but unfortunately the patient's condition continued to deteriorate and, ultimately, the decision was made by her family to withdraw care.

## DISCUSSION

Upon further discussion with her health care agent, it was discovered that the patient had undergone a number of previously undisclosed cosmetic procedures. An unreported Botox injection was presumed to have interfered with neuromuscular function testing on the facial nerve. We were unable to confirm this with the patient, as she died several days later.

This study exemplifies the risk of suboptimal neuromuscular blockade when titrated to facial nerve stimulation testing. Thus, neuromuscular function should be monitored with the ulnar nerve whenever possible, as previously expressed in the literature.^8^ In addition, this highlights the primacy of monitoring all aspects of the patient's condition and avoid overreliance upon a single clinical parameter, in this case the train-of-four monitoring, particularly in light of contradictory clinical information. As the prevalence of Botox procedures continues to rise, all patients, regardless of age or youthful appearance, should be preoperatively screened for prior Botox use. Furthermore, we recommend that plastic surgeons educate patients about disclosing information regarding all cosmetic procedures to prospective doctors no matter how minor they may view it.

To conclude, adequate documentation of Botox history is a key component to ensuring optimal medical care and ensuring patient safety.
